# A Registration Method Based on Ordered Point Clouds for Key Components of Trains

**DOI:** 10.3390/s24248146

**Published:** 2024-12-20

**Authors:** Kai Yang, Xiaopeng Deng, Zijian Bai, Yingying Wan, Liming Xie, Ni Zeng

**Affiliations:** School of Physical Science and Technology, Southwest Jiaotong University, Chengdu 610031, China

**Keywords:** point cloud registration, ordered point cloud, 2.5D point cloud, image feature matching

## Abstract

Point cloud registration is pivotal across various applications, yet traditional methods rely on unordered point clouds, leading to significant challenges in terms of computational complexity and feature richness. These methods often use k-nearest neighbors (KNN) or neighborhood ball queries to access local neighborhood information, which is not only computationally intensive but also confines the analysis within the object’s boundary, making it difficult to determine if points are precisely on the boundary using local features alone. This indicates a lack of sufficient local feature richness. In this paper, we propose a novel registration strategy utilizing ordered point clouds, which are now obtainable through advanced depth cameras, 3D sensors, and structured light-based 3D reconstruction. Our approach eliminates the need for computationally expensive KNN queries by leveraging the inherent ordering of points, significantly reducing processing time; extracts local features by utilizing 2D coordinates, providing richer features compared to traditional methods, which are constrained by object boundaries; compares feature similarity between two point clouds without keypoint extraction, enhancing efficiency and accuracy; and integrates image feature-matching techniques, leveraging the coordinate correspondence between 2D images and 3D-ordered point clouds. Experiments on both synthetic and real-world datasets, including indoor and industrial environments, demonstrate that our algorithm achieves an optimal balance between registration accuracy and efficiency, with registration times consistently under one second.

## 1. Introduction

Point cloud registration plays a crucial role in many application scenarios, including 3D reconstruction [1,2,3], building information modeling (BIM) [4,5], autonomous driving [6,7,8], and robotics [9]. The goal of point cloud registration is to estimate the optimal rigid transformation between two point clouds to accurately align them under different viewpoints. In recent years, with the rapid development of sensor technology, point cloud data can be easily obtained using devices such as radar, structured light sensors, depth cameras, stereo cameras, and 3D sensors. Based on the characteristics of point clouds, they can be categorized as unordered or ordered point clouds.

Traditional point cloud registration methods can be divided into optimization-based methods and feature-based methods. Optimization-based methods [10,11,12,13,14,15,16,17,18] typically iteratively search for corresponding points and calculate transformation matrices, which can easily fall into local optimal solutions. Feature-based methods [19,20,21,22,23,24,25] usually extract keypoints from point clouds for registration. In recent years, with the development of deep learning, some methods [26,27,28,29,30] have used deep neural networks to estimate corresponding points. However, these methods are all based on unordered point clouds, where the relationships between points are unknown, making it time-consuming to find corresponding points or calculate feature points. Additionally, from a certain perspective, it may be impossible to capture a specific part of an object, making it difficult to obtain accurate and detailed local information. Typically, nearest neighbor search methods (KNNs) are used to obtain local information about points, but acquiring neighborhood information at object boundaries may cause the neighboring points to not extend beyond the object boundary, resulting in less local information and the inability to utilize information beyond the object boundary. The KNN method is also susceptible to uneven point cloud density, resulting in varying receptive fields for dense and sparse regions, leading to unstable algorithm performance. Furthermore, the computational complexity of the KNN algorithm is $O\left(n^{2}\right)$, posing challenges when dealing with large point cloud datasets.

Recently, in industrial environments, the use of 3D cameras or three-dimensional reconstruction methods [1,2,3] to acquire point clouds has emerged. This method of obtaining point clouds ensures that the positions of points are known, thus categorizing them as ordered point clouds. Specifically, both 3D cameras and three-dimensional reconstruction techniques can capture the 2D visual information (images) and 3D point clouds of objects. The 2D coordinates of the point cloud and the pixel coordinates of the image are correspondingly related, allowing the world coordinates of a point to be obtained through the pixel coordinates of the image. When seeking local information of a point, its pixel coordinates can be used to acquire its neighboring points. This neighborhood point search method allows points beyond the object’s boundaries, ensuring that local information includes boundary details and remains unaffected by point cloud density, with a complexity of O(n). Consequently, ordered point clouds offer superior advantages over unordered point clouds, exhibiting enhanced performance in point cloud registration.

Feature-based registration techniques typically entail the extraction of keypoints from point clouds, the computation of feature descriptors, and the subsequent implementation of robust estimation procedures. An unsuccessful outcome in any of these phases can result in a failed registration process. The identification of effective keypoints for point cloud registration poses a considerable challenge. Moreover, the extraction of keypoints is a time-intensive task. Some approaches [20,25] leverage local neighborhood points to establish a covariance matrix, conduct feature decomposition, and utilize eigenvalues and eigenvectors to ascertain if a point resides at a corner for keypoint determination. In this study, a method is proposed where the local features of points are directly computed, and corresponding point pairs are identified by assessing feature similarities, thereby obviating the necessity for keypoint extraction. Leveraging the ordered structure of the point cloud enables swift computation of local features for all points within the cloud.

Due to the correlation between ordered point clouds and their 2D images, we also employ image feature-matching techniques for point cloud registration. The specific procedure involves computing keypoints in images from different perspectives, utilizing brute-force matching to pair 2D keypoints, deriving two sets of corresponding 3D points based on their pixel coordinates, subsequently applying robust estimation methods such as RANSAC [31], BANSAC [32], and [33] to eliminate incorrect correspondences, and ultimately calculating the transformation matrix.

## 2. Related Works

### 2.1. Image Feature Matching

The SIFT algorithm [34] employs image pyramids and scale-space analysis to extract feature points, determine their orientation, and create descriptions based on scale and orientation, providing a robust foundation for feature matching. In contrast, while the FAST algorithm [35] is highly efficient in extracting feature points, it lacks the capability to determine orientation, resulting in insufficient descriptors for tasks requiring rotation invariance. The BRIEF algorithm [36], which addresses storage and computational efficiency by producing binary descriptors, simplifies distance calculations but is highly sensitive to orientation changes, thus lacking rotational invariance. To mitigate this, the ORB algorithm [37] extends FAST by incorporating an orientation estimation step and uses a modified, orientation-aware version of BRIEF, providing rotationally invariant descriptors with efficiency similar to FAST. Finally, BEBLID [38] utilizes integral images to compute grayscale differences between pairs of square regions across two images. By employing an enhanced feature selection process, BEBLID produces robust, compact descriptors that further improve feature-matching efficiency.

Some scholarly works leverage deep neural networks for image matching. One notable example is SuperPoint  [39], a self-supervised neural network designed for detecting keypoints and computing descriptors. Another significant network is SuperGlue [40], which focuses on feature matching by predicting the matching relationships among the identified keypoints. Additionally, LightGlue [41] enhances the efficiency and accuracy of feature-matching processes.

Image feature matching can estimate a homography matrix, while point cloud feature matching can calculate a rigid transformation. As previously discussed, the 2D coordinates of 3D point clouds and images are correlated. Our approach employs image feature matching to establish correspondence between two point clouds and calculate the transformation matrix.

### 2.2. Point Cloud Keypoints

ISS [20] computes the covariance matrix of a point and performs feature decomposition. If the ratio of two adjacent eigenvalues is below the threshold, this point is classified as a keypoint. Harris3D [25] extends the corner detection method from 2D images to 3D space. While Harris2d [42] utilizes image gradients to construct a covariance matrix, Harris3d employs the surface normal vector *Z* and two perpendicular directions, *X* and *Y*, to construct the covariance matrix. Notably, a significant variation in point cloud density occurs when traversing the *X* and *Y* directions at corner locations. SIFT3d [43] computes the Gaussian difference values for all candidate keypoints and discards those with Gaussian difference values below a specified threshold relative to the maximum value. NARF [44] focuses on extracting keypoints from 3D depth images. The process involves initial edge extraction, followed by the computation of a score representing the local surface variation for each point in the range image, as well as the identification of the primary direction of this variation. Subsequently, a non-maximum suppression step is applied to obtain the final keypoints.

The USIP [45] algorithm is capable of identifying highly repetitive and precisely located keypoints within 3D point clouds in an unsupervised fashion. RSKDD [46] introduced a straightforward attention mechanism for aggregating neighboring points to generate keypoints directly. Ref. [47] involves an unsupervised network for generating 3D point cloud keypoints, taking into account the probability distribution and spatial arrangement of keypoints. Nonetheless, these deep learning methods are limited in their ability to generalize to various scenes, particularly industrial environments.

These methods complete registration by defining keypoints, but it is challenging to define what keypoints are effective for registration. In this paper, we directly compare the feature similarity between two points to determine matching points without extracting keypoints, thereby removing a step that may affect registration performance.

### 2.3. 3D Feature Descriptors

The computation of point pair features (PPFs) [48] involves determining the angles formed by the normal vectors of two given points, the angle between the normal vector and the vector connecting these points, and the distance separating the two points. The PFH [49] method establishes a local coordinate system and computes quaternion features between two points, with a computational complexity of O(n(k×(k−1)/2). The fast point feature histograms (FPFHs) [19] algorithm does not measure the distance between points because the distance is influenced by the density of the point cloud. The computational complexity of FPFH is O(nk). SHOT [50] computes the covariance matrix to establish a local coordinate system, whereby the spatial neighborhood of points is divided into several subspaces. Subsequently, the normal characteristics of each point within the subspace are statistically encoded into histograms. These histograms from each subspace are then combined to generate a three-dimensional descriptor.

PPPFNet [26] computes a 3D representation as a collection of PPF combined with points and normals within a local vicinity. Riga [13] employed PPF signatures to learn rotation-invariant and globally aware descriptors. RoReg [33] designed a novel-oriented descriptor and estimates local rotations. Evidently, these deep descriptors offer enhanced distinctiveness for precise matching. In contrast, our method focuses on computing geometric features that suffice for accurate point correspondence determinations.

The process of querying local neighborhood points using the KNN method or the neighborhood balls method is characterized by high complexity in computing pairwise features among these neighboring points and is susceptible to the uneven density of point clouds. This study involves obtaining neighboring points through pixel coordinate indexing.

## 3. Method

### 3.1. Overview

The point cloud obtained from 3D or depth cameras requires consideration of both 2D and 3D positions for each point. The 2D position pertains to the pixel’s location in the image, while the 3D position refers to its precise location within the world coordinate system. In this context, the 2D distance denotes the measurement between pixels in the image, while the 3D distance refers to the Euclidean distance between the corresponding 3D points.

This study evaluates two algorithms. The first approach leverages the ordered characteristics of point clouds for registration, whereas the second approach uses the correspondence between 2D visual images and 3D point clouds, facilitated by image feature matching, to achieve registration. For clarity, these algorithms are designated as Algorithms 1 and 2.

### 3.2. Transformation Matrix Computation from Ordered Point Clouds

First, we offer a brief overview of the proposed approach that computes the transformation matrix directly from ordered point clouds. We initially calculate features for all points in the source point cloud by obtaining neighborhood information through pixel coordinate indexing. Next, we iteratively compute features for each point in the target point cloud and compare the similarity with the characteristics of all points in the source point cloud. If the similarity of two points satisfies the criteria, the correspondence is kept. The process continues until the desired number of corresponding points is reached. The algorithm can be viewed in Algorithm 1.

**Figure 1:**
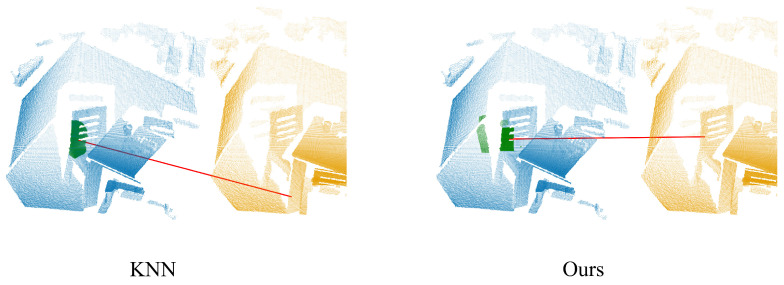
The difference between the KNN query method and our approach. The KNN query method constrains neighboring points to fall within the object boundary, whereas our approach allows neighboring points to extend beyond the object boundary. The blue and yellow point clouds denote the source and target, respectively, with green indicating the local neighborhood points and the red line segment denoting the matching point. Our method can accurately match corresponding points when only utilizing local features.

Once the pixel coordinates of a query point are identified, neighborhood points are obtained based on spatial relationships between pixels. The differences between the proposed neighborhood search method and the KNN search method are illustrated in Figure 1. It is evident that when the query point lies at the object boundary, our approach provides a more comprehensive set of neighborhood information, allowing neighborhood points to extend beyond the object boundary. In contrast, KNN query results are constrained to points within the object boundary. By extending neighborhood points beyond object boundaries, our approach facilitates the extraction of more detailed local features. Through such a neighborhood query method, the local features are more abundant. As can be seen from Figure 1, the corresponding points can be correctly matched only by taking the Euclidean distance from the neighborhood point to the central point as their local features.

**Figure 2:**
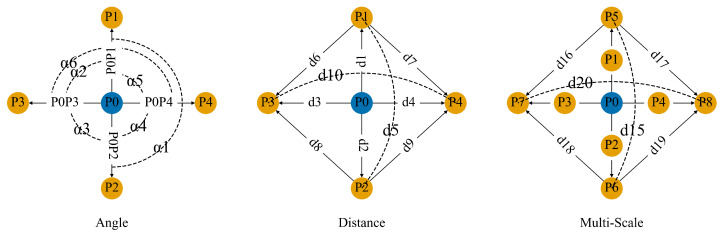
The distance and angular relationships between neighboring points and the central point. The blue represents the central point, while the yellow represents neighboring points. The multi-scale feature extraction layer consists of three layers, but for the sake of simplicity, we illustrate two layers.

The local neighborhood points of a given point $P_{0}$ are defined by the four points $P_{1},P_{2},P_{3},P_{4}$ positioned above, below, to the left, and to the right of the central point, at a 2D distance of *d* from the central point. We argue that these four points can effectively convey local information while taking into account computational efficiency. The distance and angular relationships between these neighboring points and the central point are computed to characterize the local features of the central point. These distance and angular relationships are illustrated in Figure 2.
**Algorithm 1** Transformation matrix computation from ordered point clouds**Require:** Source point cloud *P*, target point cloud *Q*, number of corresponding points *N*, curvature threshold $t_{c}$, dissimilarity threshold $t_{s}$**Ensure:** Transformation matrix *T*1:Initialize parameters for normal vector computation, feature matching, and thresholds $t_{c}$ and $t_{s}$.2:Compute normal vectors and curvatures for both point clouds *P* and *Q*. Calculate all point features for the source point cloud *P*.3:n←04:**while** n<N **do**5:    Randomly select a point $Q_{i}$ from the target point cloud *Q*.6:    **if** curvature($Q_{i}$) $<t_{c}$ **then**7:        **continue**8:    **end if**9:    Calculate the features of $Q_{i}$ and compare these with features of all points in the source point cloud *P* to identify the most similar point, forming a corresponding pair.10:    **if** dissimilarity between points $>t_{s}$ **then**11:        n←n+112:        **continue**13:    **end if**14:    Save corresponding pair.15:    n←n+116:**end while**17:Apply the RANSAC algorithm to eliminate erroneous correspondences.18:Compute the transformation matrix *T* using singular value decomposition (SVD).

The local characteristics of a point can be expressed in terms of the distance and angle between the central point and its neighboring points. The angle formed by two vectors, $P_{0}P_{1}$ and $P_{0}P_{3}$, can be mathematically represented as follows:

(1) $$\theta =\cos^{-1}\left(\frac{\left(P_{1}-P_{0}\right)\cdot \left(P_{3}-P_{0}\right)}{∥P_{1}-P_{0}∥\cdot ∥P_{3}-P_{0}∥}\right)$$

The 3D distance between two points $P_{0}$ and $P_{1}$ can be denoted as $\|P_{1}-P_{0}\|$. The local characteristics of a point can be represented by a 6-dimensional angular feature or a 10-dimensional distance feature.

We also computed the PPF [48] and PFH [49] features of the four neighboring points and the central point. However, we focus exclusively on the features between the central point and neighboring points, omitting the features among neighboring points. The dimensionality of the local features is 16, comprising four pairs of point-wise features. Each pair of point features consists of four dimensions, including three angular features and the Euclidean distance between the two points.

The structured arrangement of point clouds facilitates the efficient calculation of normal vectors and curvature. The normal vector of a point can be represented by the normal vectors of the planes within its local neighborhood. We select all neighboring points that lie within a square of size *s* with $p_{i}$ at the center. The computation of the normal vector can be achieved by minimizing an objective function, as follows:

(2) $$S=\sum_{i=1}^{n}{\left(ax_{i}+by_{i}+cz_{i}\right)}^{2}$$

The vector (a,b,c) acts as the normal vector to the plane. The coordinates of a neighboring point are $\left(x_{i},y_{i},z_{i}\right)$. The number of points required to estimate a normal vector is *n*. Taking the partial derivatives with respect to *a*, *b*, and *c* yields the following:

(3) $$\left(\begin{array}{ccc} \sum x_{i}^{2} & \sum x_{i}y_{i} & \sum x_{i}z_{i}\\ \sum x_{i}y_{i} & \sum y_{i}^{2} & \sum y_{i}z_{i}\\ \sum x_{i}z_{i} & \sum y_{i}z_{i} & \sum z_{i}^{2} \end{array}\right)\left(\begin{array}{c} a\\ b\\ c \end{array}\right)=\left(\begin{array}{c} 0\\ 0\\ 0 \end{array}\right)$$

The eigen decomposition is performed on the coefficient matrix on the left side of the equation. The eigenvector corresponding to the smallest eigenvalue serves as the normal vector, with ambiguity removed through sign consistency. The eigenvalues are denoted as $\lambda_{1}$, $\lambda_{2}$, and $\lambda_{3}$, arranged in descending order. The curvature is calculated as $\frac{\lambda_{3}}{\lambda_{1}+\lambda_{2}+\lambda_{3}}$.

Distinguishing entities with similar characteristics, such as points on a plane, is challenging. One approach to address this challenge involves computing multi-scale features to enhance local receptive fields, utilizing points in the surrounding areas in all four directions as neighboring points to the center point. The 2D distances between multi-scale neighboring points and the center point correspond to pixel distances of a,3a,5a. The smaller scale neighboring points are denoted as $P_{1},P_{2},P_{3},P_{4}$, while the larger scale neighboring points are represented as $P_{5},P_{6},P_{7},P_{8}$. The local contextual relationships of multi-scale feature extraction methods are illustrated in Figure 2.

To address the issue of feature ambiguity, one approach, in addition to computing multi-scale features, involves removing points located within the planar region. Specifically, points with curvatures below a certain threshold are excluded, and therefore, their features are not considered in the calculations.

In feature matching, cosine similarity is employed. After identifying the most similar features, their dissimilarity is calculated to determine whether to retain this pair of points. The dissimilarity is expressed as follows:

(4) $$E=\frac{1}{d}\times \sum_{i=1}^{d}S_{i},$$

where

(5) $$S_{i}=\left\{\begin{array}{cc} 0 & \mathrm{if}\;|P_{i}-Q_{i}|\;<0.01\\ 1 & \mathrm{else} \end{array}\right.$$

The dimensionality of the local features is denoted by *d*. The number of feature differences exceeding 0.01 is calculated, and if their ratio surpasses 0.5, the pair of points is excluded from selection.

### 3.3. Transformation Matrix Computation from Images and Point Clouds

As previously mentioned, the 2D visual information and 3D point cloud data obtained from 3D cameras exhibit a strong correlation. We use image feature extraction and matching to establish 3D point correspondences. Specifically, we utilize SIFT to extract keypoints and descriptors from 2D images and employ a brute-force matching method to establish 2D point correspondences. Due to the direct correspondence between 2D visual images and 3D point cloud data, we can derive 3D points from 2D coordinates, facilitating the establishment of 3D point correspondences. We expand the range of 2D keypoints to obtain more 3D keypoints. Subsequently, the transformation matrix is computed using a robust estimation method to effectively eliminate erroneous matches. Please see Algorithm 2 for further details.
**Algorithm 2** Transformation matrix computation from images and point clouds**Require:** Image $I_{1}$, Image $I_{2}$, Point cloud *P*, Point cloud *Q***Ensure:** Transformation matrix *T*1:Initialize relevant parameters for SIFT, matching, and RANSAC.2:Extract keypoints and descriptors from images $I_{1}$ and $I_{2}$ using SIFT.3:Perform brute-force matching to identify corresponding 2D points between $I_{1}$ and $I_{2}$.4:Retrieve the corresponding 3D points in *P* and *Q* based on matched 2D coordinates.5:Apply the RANSAC algorithm to exclude erroneous matching pairs.6:Compute the transformation matrix *T* using singular value decomposition (SVD).

While the method of extracting keypoints and descriptors from 2D images can achieve point cloud registration, it overlooks the valuable geometric information inherent in point clouds. Our approach leverages Algorithm 2 for 2D keypoint extraction, followed by Algorithm 1 to compute descriptors directly from the ordered point clouds, thereby validating the effectiveness of Algorithm 1. By utilizing 2D keypoints in conjunction with either 2D features or 3D features, we exploit the correspondence between ordered point clouds and 2D images, enhancing registration accuracy and robustness.

**Figure 3:**
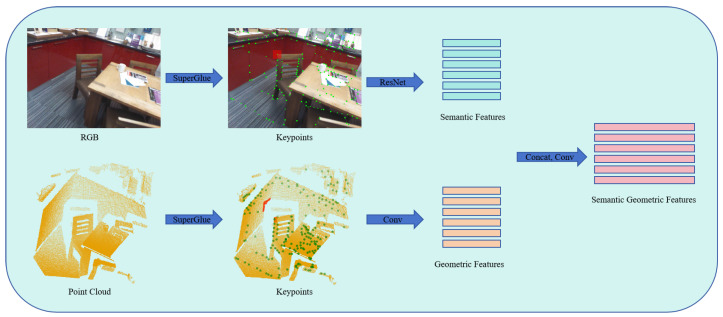
Fusion of semantic and geometric features. The pretrained SuperGlue network is employed to extract keypoints, which are expanded in both 2D images and 3D point clouds to generate more keypoints, as highlighted by the red rectangle in the second column. Semantic features are learned by ResNet with gradient stopping applied, as depicted in the top section. Geometric features are computed using neighboring points within a local window, as outlined in Algorithm 1, and learned by a multi-layer perceptron (MLP), as depicted in the bottom section. Finally, these semantic and geometric features are concatenated and further processed by an MLP to achieve feature fusion.

To further enhance registration performance, we introduce a neural network that fuses 2D semantic features with 3D geometric features. Specifically, we employ the pretrained SuperGlue network [40] to extract keypoints, expanding the range of keypoints in both 2D images and 3D point clouds. ResNet is utilized to extract semantic features from 2D images, with gradient stopping applied to prevent backpropagation into the feature extraction process. Local geometric features are initially computed using neighboring points within a local window, as outlined in Algorithm 1, and then learned by a multi-layer perceptron (MLP). Leveraging the correspondence between 2D images and 3D point clouds, we merge the semantic and geometric features through convolutional operations, as depicted in Figure 3. Triplet loss is employed to train the descriptors, ensuring that nearby points exhibit similar features while distant points exhibit greater feature differences, thereby enhancing registration accuracy and robustness. This multimodal fusion approach leverages the strengths of both semantic and geometric information, resulting in improved registration performance.

### 3.4. Evaluation

Based on ordered point clouds, a new evaluation metric for point cloud registration is proposed. When acquiring point cloud data with a 3D camera, the projection of the point cloud into the pixel coordinate system depends on camera intrinsic parameters. In this process, the depth *z* of the point cloud is used as its pixel value. The re-projection process is defined as follows:

(6) $$\left(\begin{array}{c} u\\ v\\ 1 \end{array}\right)\left(\begin{array}{ccc} f_{x} & f_{0} & c_{x}\\ 0 & f_{y} & c_{y}\\ 0 & 0 & 1 \end{array}\right)=\left(\begin{array}{c} x\\ y\\ 1 \end{array}\right)$$

In the pixel coordinate system, two sets of point clouds are denoted as $I_{1}$ and $I_{2}$. Upon the successful registration of the two point clouds, their pixel values at the same pixel position should exhibit minimal discrepancies. Since two point clouds are typically acquired from different viewpoints, they often do not entirely overlap. The evaluation of registration efficacy is based on the computation of differences in depth within the overlapping region of the point clouds. The overlapping regions can be denoted as $Mask=min_{z}<I_{1},I_{2}<max_{z}$. Here, $min_{z}$ and $max_{z}$ denote the minimum and maximum depth values, respectively. The registration error can be calculated as follows:

(7) $$\mathrm{Residual}=\frac{1}{U\times V}\sum_{u,v\in \mathrm{Mask}}^{U,V}\left|I_{1}\left(u,v\right)-I_{2}\left(u,v\right)\right|$$

A smaller registration residual indicates a more effective registration.

## 4. Experiment Results

Since our point cloud registration method is based on ordered point clouds, we utilized datasets such as Flyingthings3D, Monkaa, and KITTI from Scene Flow [51]. As the point clouds of adjacent frames are not subjected to rigid transformations, we employed random rotations and translations to generate pairs of point clouds for our study. We also employed the 3DMatch [52] data to demonstrate our method, which was obtained from depth sensors, including Microsoft Kinect, Structure Sensor, Asus Xtion Pro Live, and Intel RealSense. The raw point cloud data are generated by fusing fifty adjacent frames. By utilizing camera intrinsics and depth maps, we transform these datasets into point cloud images, where individual channels correspond to the *x*, *y*, and *z* coordinates of the point cloud data. Subsequently, we treat point clouds separated by 50 frames as pairs. Additionally, an experiment is conducted on the data captured by a 3D camera. The two sets of point clouds were acquired from different perspectives and time frames. The RGB 2D images, depth map, and point cloud data are synchronously captured by the Zivid 2 M70 camera, specifically engineered for high-precision machine vision tasks. The point cloud image, sized at 1944×1200, undergoes downsampling at intervals of every 5 rows and 5 columns to expedite algorithmic processing speed. The curvature threshold $t_{c}$ is 0.005, and the dissimilarity threshold for features $t_{s}$ is 0.5.

### 4.1. Ordered Point Cloud Registration Experiments

We initially conducted experiments on the Scene Flow and 3DMatch datasets, employing distance and angle features in a single-scale approach to validate the effectiveness of our method. The dimensionality of the local features for each point is established as 16. A comparative analysis was conducted between conventional point cloud registration methods, including the ICP [14] algorithm and the Harris [25] algorithm for keypoint extraction, followed by the calculation of FPFH [19] descriptors for registration purposes.

**Figure 4:**
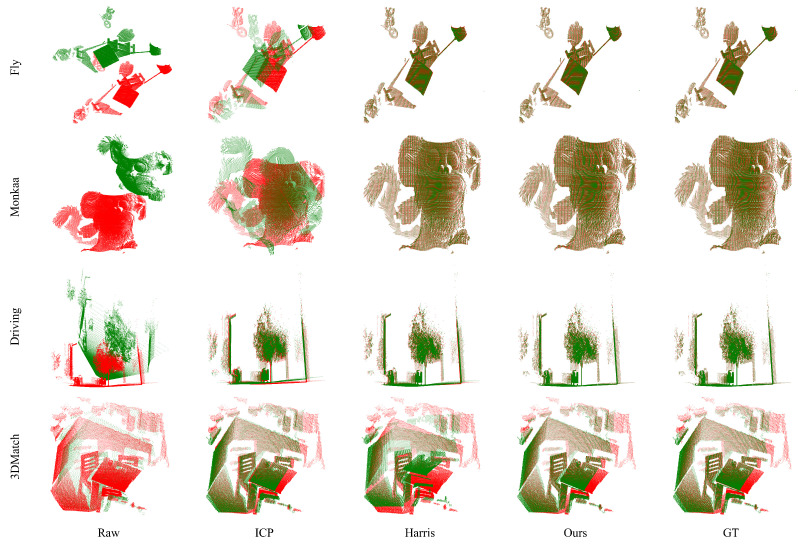
The comparison results of our method against the ICP algorithm and the Harris + FPFH algorithm. Our method exhibits superior registration performance across all datasets compared to other approaches, achieving results that closely align with the ground truth. The green color denotes the source point cloud before and after registration, while the red color indicates the target point cloud.

To evaluate the registration performance, we employed several metrics: the registration residual, nearest neighbor distance, and root mean square error (RMSE) between the calculated registration matrix and the ground truth. The results presented in Figure 4 indicate that our approach surpasses other methods regarding registration effectiveness. Furthermore, as shown in Table 1, our method displays the highest registration accuracy among the compared techniques. Additionally, it is evident that when the RMSE of registration is low, both the registration residual and the nearest neighbor distance are minimal. This further substantiates the effectiveness of the proposed evaluation metrics.

Our methodology demonstrates a high level of proficiency in point cloud registration, whether it involves aligning two point clouds obtained through rotation and translation or those captured from various perspectives and time frames. This indicates the robustness and versatility of our approach across diverse scenarios.

**Figure 5:**
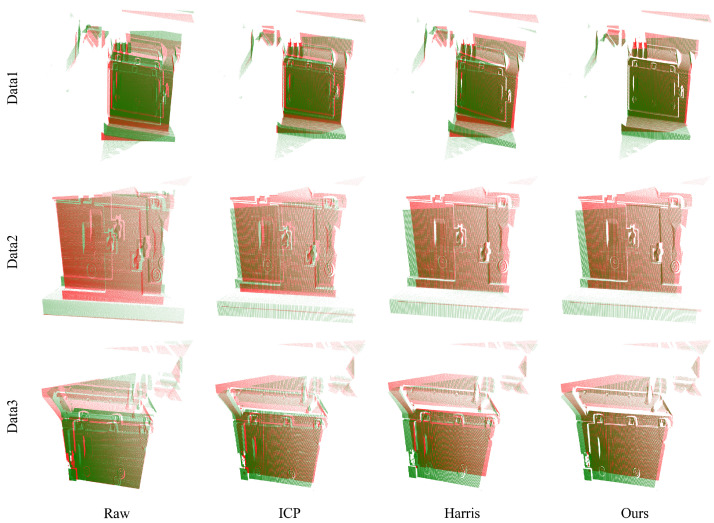
The registration performance of the point cloud acquired from the 3D camera. Our approach demonstrates superior registration effectiveness in comparison to alternative methodologies. The green color denotes the source point cloud before and after registration, while the red color indicates the target point cloud.

We employed a 3D camera to capture the same object from multiple perspectives, focusing on essential components of a train. However, due to the unknown relative positions between the two captures, a direct comparison with the ground truth is not possible. To evaluate the registration effectiveness, we employed registration residuals and nearest neighbor distances. Given that the captured object primarily consisted of flat surfaces, this presents challenges for many keypoint-based matching methods. By computing the curvature of the point cloud and pre-filtering points with curvature below a specified threshold, we performed feature matching using single-scale feature extraction techniques. As illustrated in Figure 5, our approach successfully registers the two point clouds. The high registration accuracy of our method, as shown in Table 2 and Figure 5, validates the efficacy of the proposed evaluation metrics.

As shown in Table 3, our approach utilizes the ordered structure of point clouds to derive local neighborhood points based on their 2D coordinates, facilitating rapid feature extraction. Notably, we use locality-sensitive hashing for similarity feature retrieval, which can be replaced by the Faiss library to enhance computational speed. The proposed method achieves registration times of under 1 s for all datasets.

### 4.2. Feature Enhancement Experiments

We conducted comprehensive experiments on the proposed method utilizing PPF [48] and PFH [49] features, initially focusing on single-scale features within large planar point clouds. To facilitate a more effective comparison of various feature extraction methods, we define two points as corresponding if the 3D distance between them is less than 1 mm. By randomly sampling 1024 pairs of matching points, we find that the PFH feature extraction method retrieves the highest number of inliers. To enhance the discriminative capability of features, we employ a multi-scale approach for feature extraction. The feature scales of 2, 5, and 8 represent pixel distances from the central point. Table 4 indicates that all feature extraction methods achieve registration, with the PPF method yielding the highest number of inliers.

By excluding planar points, all techniques extract a greater number of inliers than when these points are not removed, regardless of whether features are extracted using single-scale or multi-scale methods. The results are presented in Table 5. Integrating curvature into PPF produces the maximum number of inliers. Furthermore, the registration speed is significantly faster than in scenarios where planar points are not excluded.

Through experimentation, we observe that first excluding the point cloud of a planar region, followed by employing a multi-scale approach, leads to the extraction of a greater number of inliers. Furthermore, increasing the feature scale reduces feature ambiguity, enabling features to be matched more accurately; however, this may result in increased registration time.

**Figure 6:**
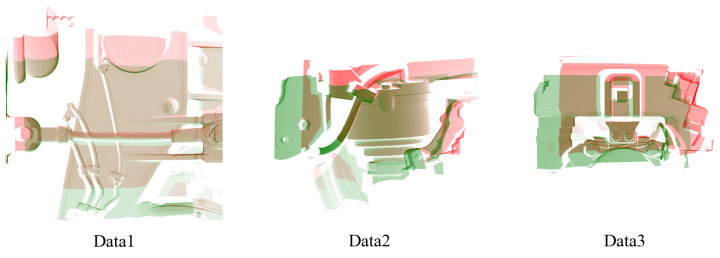
The registration results for scenes containing non-rigid objects. The green color denotes the source point cloud before and after registration, while the red color indicates the target point cloud.

**Figure 7:**
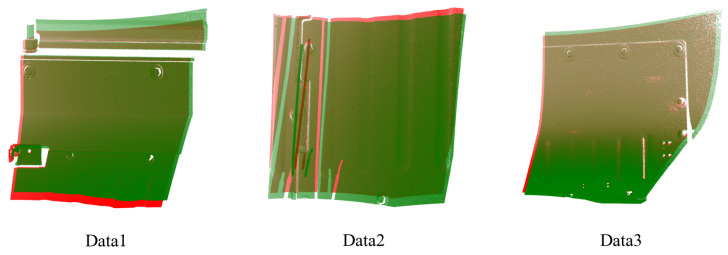
The registration results for scenes with extensive planar areas and increased noise levels. The green color denotes the source point cloud before and after registration, while the red color indicates the target point cloud.

Additionally, we tested the feature extraction method using PPF in more complex scenarios. This is illustrated in Figure 6 and Figure 7. Figure 6 features additional non-rigid objects in the scene, such as pipes, which present challenges in computing a rigid transformation matrix. In Figure 7, the scene consists primarily of planar regions and contains a higher level of noise. Our algorithm successfully registers two point clouds in complex scenes.

**Figure 8:**
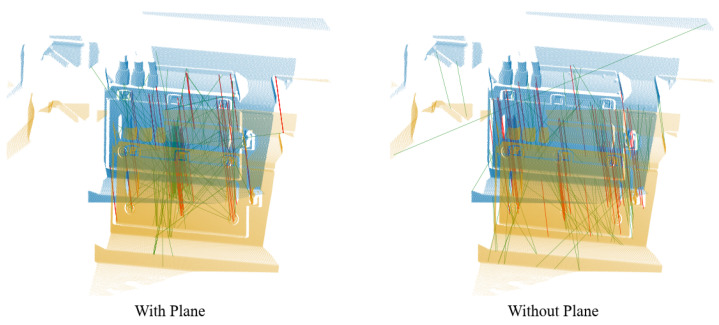
The extracted matching points. The lines in red represent the correctly matched points, whereas the incorrectly matched points are depicted in green. The label “With Plane” indicates that points on a plane are not removed, while the label “Without Plane” signifies that points on a plane have been removed.

To demonstrate the significance of removing planar points, we visualize the matching points after this removal. As illustrated in Figure 8, the number of correct matching points increases after the removal of planar points from the surface, thereby verifying the effectiveness of our method in addressing feature ambiguity and enhancing the matching of corresponding points.

### 4.3. Image-Assisted Information Registration

**Figure 9:**
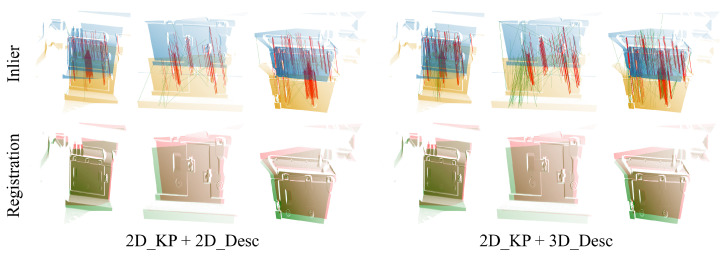
The matching points and registration results. The left section illustrates the matching and registration results obtained with the SIFT algorithm, while the right section depicts results obtained using a combination of the SIFT algorithm for keypoint extraction and Algorithm 1 for descriptor computation. The top section displays both correctly and incorrectly matched points, where red lines indicate correctly matched points and green lines indicate incorrectly matched points. The bottom section presents the registration results.

To investigate the correlation between the 2D visual information captured by our 3D camera and the 3D point cloud, we extract keypoints and perform feature matching directly on two 2D images designated for registration. Subsequently, we employ the 2D coordinates of the matched points to retrieve their corresponding 3D points. We employ the SIFT [34] algorithm to compute the feature points and descriptors of 2D images while conducting brute-force matching. Using the 2D coordinates of the matching points, we retrieve the corresponding 3D points. The RANSAC algorithm is subsequently employed to eliminate mismatched points. The extracted inliers and registration results are illustrated on the left side of Figure 9.

Many algorithms for extracting keypoints from point clouds encounter difficulty in accurately detecting corner points, such as those present on bolts in train systems. Conversely, methods for extracting keypoints from 2D images are widely established. This research incorporates a 2D keypoint extraction technique to identify keypoints, which are then used to reference 3D points according to their pixel coordinates. The subsequent step involves employing the algorithm detailed in Section 3.2 to identify corresponding points. By extending the identified 2D keypoints within the nearby pixel vicinity, additional 3D keypoints are obtained. The findings, illustrated on the right side of Figure 9, showcase the successful alignment achieved through 2D keypoints, thus affirming the effectiveness of Algorithm 1.

The application of a 2D keypoint extraction method facilitates the identification of critical areas, thereby eliminating the need to exclude planar region points using curvature thresholds, as previously mentioned. The correspondence between 2D visual information and 3D point clouds supports the successful implementation of this registration approach. Moreover, the feature extraction and matching process for 2D images is highly efficient, enabling registration on undownsampled data to be completed in approximately one second.

**Figure 10:**
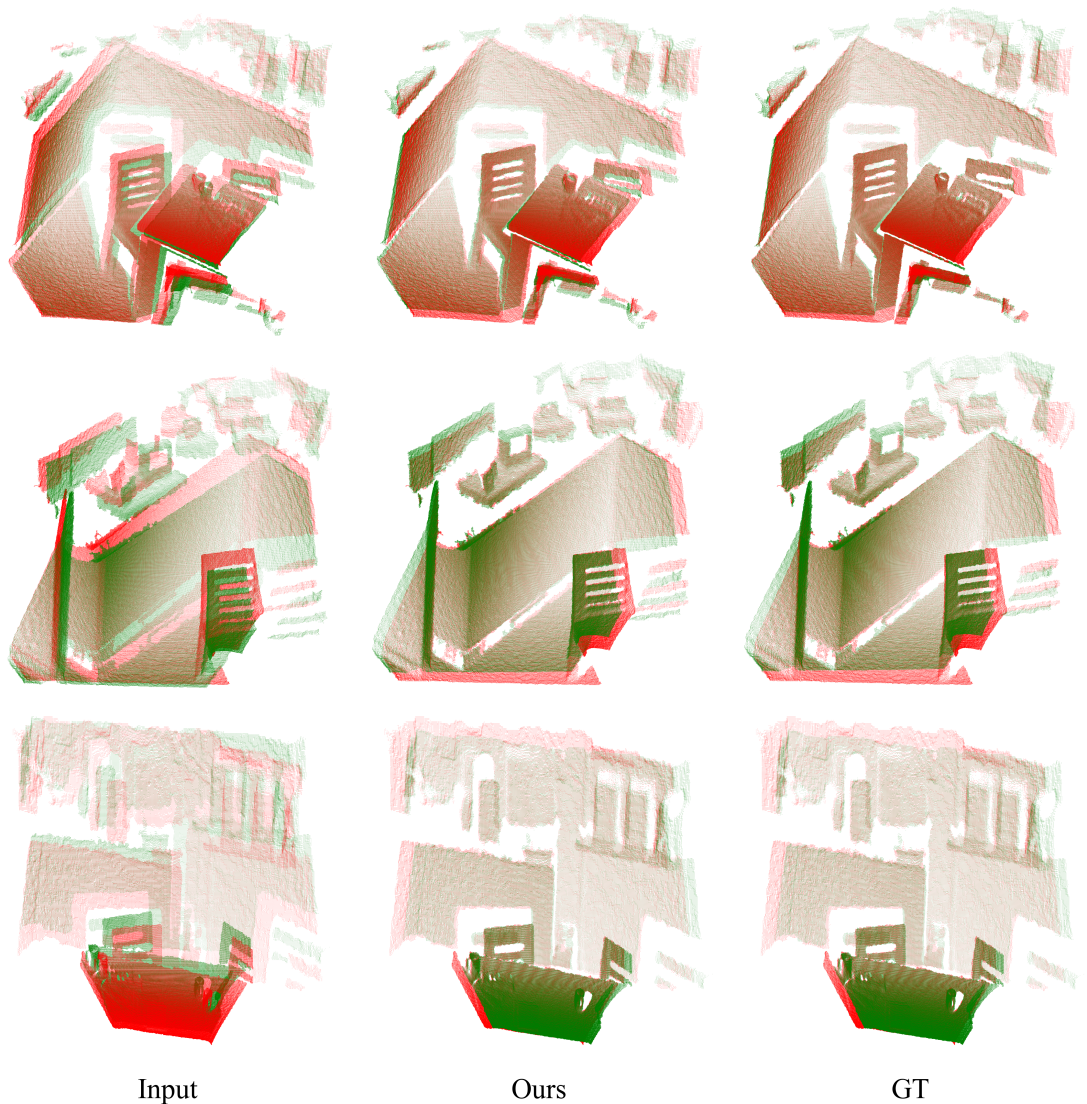
Registration results for semantic-geometric features. The red points represent the target point cloud, while the green points depict the source point cloud.

The discussion previously emphasized individual use of 2D or 3D features. Our focus now shifts to combining 2D semantic features and 3D geometric features through deep learning methods. SuperGlue [40] is utilized to extract keypoints for learning semantic-geometric features via neural networks. The registration outcomes are depicted in Figure 10. Our method demonstrates successful integration of 2D semantic and 3D geometric features on the 3DMatch dataset, utilizing multimodal features for effective point cloud registration.

## 5. Discussion

In this paper, we propose a registration method based on ordered point clouds. Feature-based point cloud registration typically consists of extracting keypoints, establishing correspondence, and robustly estimating transformation parameters. Any step failure could affect the performance of the final result. Some technologies, such as ISS, Harris, and NARF, as well as methods based on deep learning, are commonly used to extract keypoints; however, the extracted keypoints may not be appropriate for registration. Our proposed algorithm directly matches corresponding points without the need for keypoint extraction. However, accurate correspondence is crucial for registration, and our proposed method may still produce a large number of outliers. Some deep learning techniques extract the features of points through data-driven methods, enabling more accurate matching with fewer outliers.

For matching points with a large number of outliers, robust estimation techniques, such as RANSAC [31], BANSAC [32], and [33], are needed to remove incorrect matches. To improve registration accuracy, the scale of feature extraction and the number of points can be increased, but this will undoubtedly increase registration time. Therefore, to balance the accuracy and speed of registration, this paper uses a smaller feature scale and fewer matching points. Nonetheless, there are existing algorithms with faster feature search methods, such as the one by Faiss [53].

Additionally, we use image feature-matching technology to register ordered point clouds. In future work, we can integrate image features into our methods to extract distinctive features and we can also integrate the feature extraction method from this paper with deep learning technology to generate more accurate matching points. Furthermore, when a 2D keypoint is identified in regions with significant changes in texture, color, illumination, or other characteristics, its corresponding 3D point in the point cloud may not necessarily be situated at object boundaries, corners, or other distinctive features, potentially resulting in the 3D point not being classified as a keypoint. It is worth noting that our proposed method is only applicable to ordered point clouds, such as those obtained by 3D cameras, depth sensors, 3D reconstruction, and other methods.

## 6. Conclusions

In this paper, we propose a registration method based on ordered point clouds. The method we propose eliminates the need for detecting keypoints, opting instead to directly extract features and subsequently perform feature matching to identify corresponding points, thus mitigating the impact of unstable keypoint extraction on registration performance. Experimental validation conducted on both public datasets and those captured by our 3D camera confirms the effectiveness of the proposed approach. Our method exhibits high accuracy and speed, employing a multi-scale approach and filtering out points with low curvature to extract the maximum number of inliers. The experiment on image information-aided registration demonstrates the correspondence between ordered point clouds and 2D images, highlighting the effectiveness of leveraging semantic information from images to enhance point cloud registration.

## Figures and Tables

**Table 1:** The comparison of various methods across different datasets. The Residual metric, proposed in this paper, serves as an evaluation measure for registration efficacy, where "Nearest Dist" denotes the nearest neighbor distance and “RMSE” represents the root mean square error. Our approach exhibits superior registration performance across all datasets. The unit of measurement is meters.

Dataset	ICP			Harris + FPFH			Ours		
	Residual	Nearest Dist	RMSE	Residual	Nearest Dist	RMSE	Residual	Nearest Dist	RMSE
Flyingthings3d	1.2664	0.5921	5.3654	0.0583	0.0321	0.1043	0.0389	0.0068	0.0154
Monkka	1.0554	0.5322	1.9162	0.0066	0.0007	0.0024	0.0061	0.0014	0.0041
Kitti	2.4684	1.1347	2.4779	0.5641	0.0874	0.1927	0.3472	0.0052	0.0010
3DMatch	0.0619	0.0453	0.0830	0.2709	0.1053	0.5255	0.0211	0.0090	0.0238

**Table 2:** The accuracy of point cloud registration obtained from 3D cameras. Our approach demonstrates superior precision in comparison to alternative methodologies. The unit of measurement is millimeters.

Dataset	ICP		Harris + FPFH		Ours	
	Residual	Nearest Dist	Residual	Nearest Dist	Residual	Nearest Dist
Our’s data1	9.6624	4.7256	29.3232	16.1268	1.1107	6.2191
Our’s data2	11.8811	2.9640	21.4412	15.7987	1.3917	0.7366
Our’s data3	10.0854	2.9145	17.0912	11.8712	1.4416	0.6975

**Table 3:** The efficient evaluation of our methodology. Our methodology demonstrates the highest registration speed across all datasets, achieving a registration time of less than one second.

Dataset	ICP	Harris + FPFH	Ours
Flyingthings3d	0.1971	0.3462	0.1247
Monkka	7.5618	0.3499	0.3245
Kitti	7.3183	0.3333	0.1556
3DMatch	1.6677	0.3561	0.4203
Our’s data1	34.0493	2.5479	0.8243
Our’s data2	23.8222	1.3577	0.8741
Our’s data3	23.4809	1.4815	0.8156

**Table 4:** The evaluation results for different methods utilizing single-scale and multi-scale techniques while retaining points on a plane. When extracting features at a single scale, the PFH method yields the highest number of inliers, while the PPF method, when utilizing a multi-scale approach, is capable of extracting the greatest number of inliers. The distance unit used is millimeters, and the time unit is seconds. The best outcomes are underlined.

	Single Scale				Multi Scale			
Method	Residual	Nearest Dist	Inlier	Time	Residual	Nearest Dist	Inlier	Time
Dist.	1.1686	1.0509	1.61%	1.61	1.0958	0.9921	4.45%	3.17
Angle	7.4240	4.5882	0.29%	1.31	1.1265	1.0551	5.03%	2.59
Dist+Angle	1.1689	1.0405	1.41%	1.86	1.1199	1.0413	5.26%	4.56
PPF	1.0378	1.0638	3.22%	1.67	1.1458	1.0081	8.91%	4.24
PPF+Curv	1.2409	1.0197	2.92%	1.46	1.1381	1.0821	4.85%	7.96
PFH	1.1672	1.0634	4.13%	1.27	1.0807	1.0239	7.71%	3.35
PFH+Curv	1.3159	1.0988	2.73%	1.23	1.1452	1.0467	7.72%	7.44

**Table 5:** Following the removal of planar points, the registration results for feature extraction using single-scale and multi-scale methods are presented. Incorporating curvature in the PPF method facilitates the extraction of the maximum number of inliers when utilizing both single-scale and multi-scale feature extraction methods. The distance unit used is millimeters, and the time unit is seconds. The best outcomes are underlined.

	Single Scale				Multi Scale			
Method	Residual	Nearest Dist	Inlier	Time	Residual	Nearest Dist	Inlier	Time
Dist.	1.0962	1.0083	5.19%	0.73	1.0861	1.0297	11.28%	0.91
Angle	1.1577	1.0901	2.34%	0.69	1.0655	1.0283	11.03%	0.85
Dist+Angle	1.1059	1.2686	5.13%	0.75	1.1010	1.0575	11.43%	1.16
PPF	1.1007	0.9863	8.07%	0.79	1.0682	0.9911	11.85%	1.63
PPF+Curv.	1.0781	1.0382	8.21%	0.91	1.0832	1.0564	13.46%	3.18
PFH	1.0748	1.0458	5.66%	0.73	1.1905	1.0841	9.23%	1.27
PFH+Curv.	1.0863	1.0126	6.46%	0.73	1.0821	1.0285	11.57%	2.61

## Data Availability

The Scene Flow dataset is available at https://lmb.informatik.uni-freiburg.de/resources/datasets/SceneFlowDatasets.en.html, accessed on 1 March 2024. The 3DMatch dataset is available at 3DMatch https://3DMatch.cs.princeton.edu/, accessed on 1 March 2024. Our dataset will be made available upon request.
